# Comparative genomics of pathogenic *Leptospira interrogans* serovar Canicola isolated from swine and human in Brazil

**DOI:** 10.1590/0074-02760170119

**Published:** 2018-02

**Authors:** Luisa Z Moreno, Fabiana Miraglia, Frederico S Kremer, Marcus R Eslabao, Odir A Dellagostin, Walter Lilenbaum, Julio C Freitas, Silvio A Vasconcellos, Marcos B Heinemann, Andrea M Moreno

**Affiliations:** 1 Universidade de São Paulo, Faculdade de Medicina Veterinária e Zootecnia, Laboratório de Epidemiologia Molecular e Resistência a Antimicrobianos, São Paulo, SP, Brasil; 2 Universidade Federal Fluminense, Departamento de Microbiologia e Parasitologia, Laboratório de Bacteriologia Veterinária, Niterói, RJ, Brasil; 3 Universidade Federal de Pelotas, Centro de Desenvolvimento Tecnológico, Pelotas, RS, Brasil; 4 Universidade Estadual de Londrina, Departamento de Medicina Veterinária Preventiva, Londrina, PR, Brasil

**Keywords:** L. interrogans, serovar Canicola, genomics, plasmid

## Abstract

*Leptospira interrogans* serovar Canicola is one of the most important pathogenic serovars for the maintenance of urban leptospirosis. Even though it is considered highly adapted to dogs, serovar Canicola infection has already been described in other animals and even a few human cases. Here, we present the genomic characterisation of two Brazilian *L. interrogans* serovar Canicola strains isolated from slaughtered sows (L0-3 and L0-4) and their comparison with human strain Fiocruz LV133. It was observed that the porcine serovar Canicola strains present the genetic machinery to cause human infection and, therefore, represent a higher risk to public health. Both human and porcine serovar Canicola isolates also presented sequences with high identity to the Chinese serovar Canicola published plasmids pGui1 and pGui2. The plasmids identification in the Brazilian and Chinese serovar Canicola strains suggest that extra-chromosomal elements are one more feature of this serovar that was previously unnoticed.

Leptospirosis is a re-emerging zoonosis in both developed and developing countries (Dupouey et al. 2014). *Leptospira interrogans* is the most frequently reported species worldwide and of greater importance for public health (Haake & Levett 2015, Wang et al. 2015). Many of *L. interrogans* serovars present importance for maintenance of urban leptospirosis and are described as adapted to biased reservoir hosts, such as serovar Copenhageni and rodents and serovar Canicola and dogs (Bolin 2000). Nevertheless, Hartskeerl and Terpstra (1996) already discussed the existence of variation of maintenance hosts and their respective serovars.

The *L. interrogans* serovar Canicola is one of the most important pathogenic leptospires and, although it is considered highly adapted to dogs (Schuller et al. 2015), serovar Canicola infection has already been described in swine and a few human cases (Lecour et al. 1989, Bolin 1994, Trevejo et al. 1998, Zhu et al. 2014). In Brazil, *L. interrogans* serovar Canicola has already been detected in wild and domestic dogs, cattle and swine (Freitas et al. 2004, Zacarias et al. 2008, Silva et al. 2014).

Here, we present the genomic characterisation of two Brazilian *L. interrogans* serovar Canicola strains (L0-3 and L0-4) isolated from slaughtered sows and their comparison with human strain Fiocruz LV133 (GenBank accession number AKWU2000000).

The L0-3 and L0-4 strains were isolated from liver of slaughtered sows with unknown health history (Freitas et al. 2004), and later characterised as *L. interrogans* serovar Canicola (Miraglia et al. 2013). The strains were stored in Fletcher's medium (DIFCO/USA) enriched with 15% rabbit serum and maintained in EMJH broth (DIFCO/USA) at 30°C, as part of the *Leptospira* collection of the Laboratory of Bacterial Zoonosis - University of São Paulo. The L0-4 strain is known to be virulent and commonly used in hamster virulence assays (Batista et al. 2010, Coelho et al. 2013).

Genomic DNA was extracted and purified with illustra^™^ bacteria genomicPrep Mini Spin Kit (GE Healthcare do Brasil Ltda, São Paulo, Brazil). Whole-genome sequencing was performed through Illumina^™^ Miseq platform with 300 bp paired-end library. *De novo* assembly was performed with CLC Genomics Workbench 7.5.1 (CLC Bio, Denmark) and Geneious 10.0.3 (Biomatters Ltd, Auckland, New Zealand).

The obtained contigs were ordered according to *L. interrogans* serovar Copenhageni strain Fiocruz L1-130 reference genome (GenBank accession numbers NC_005823 and NC_005824). Automatic genome annotation was performed with NCBI Prokaryotic Genome Annotation Pipeline (Tatusova et al. 2016). The basic assembly statistics and annotation features identified are summarised in Table.

**TABLE:** Assembly statistics and basic annotation features observed for porcine Brazilian *Leptospira interrogans* serovar Canicola strains

Strain	GenBank accession	Assembly statistics					Basic annotation features		
		Scaffolds	N_50_	Length	GC%	Coverage	CDS	rRNAs	tRNAs
L0-3	LIHE00000000	319	24.742	4.71 Mb	35.2	40x	3,780	4	34
L0-4	LIIY00000000	49	200.400	4.75 Mb	35.3	90x	3,899	5	37

CDS: coding sequences; GC: guanine-cytosine content (%).

L0-3 and L0-4 drafts genomes (GenBank accession numbers LIHE00000000 and LIIY00000000) present ~4.2 and 4.3 Mb for chromosome I and ~400 and 422 kb for chromosome II, respectively. BLASTn against nonredundant NCBI database was applied for the remaining contigs, that did not align with the reference, and resulted in high identity with Chinese serovar Canicola published plasmids pGui1 (GenBank accession number NC_025136) and pGui2 (GenBank accession number NC_025197) (Zhu et al. 2014).

The same analysis was performed with strain Fiocruz LV133 available contigs; the draft genome of Fiocruz LV133 strain is available at NCBI database identified as *L. interrogans* serovar Canicola isolated from human in Brazil (GenBank accession number AKWU00000000). Fiocruz LV133 strain was characterised with ~4.4 Mb for chromosome I, ~422 kb for chromosome II and also presented sequences similar to pGui1 and pGui2.

Brazilian serovar Canicola drafts genomes were compared through Artemis comparison tool (ACT) (Carver et al. 2005) and BLAST ring image generator (BRIG) (Alikhan et al. 2011). Chromosomal content presented high synteny and 99% identity at DNA level among studied strains (Fig. 1). It is also worth noting a few deletion regions, in both chromosomes, in the serovar Canicola sequences compared to the reference; these correspond mainly to transposases, hypothetical and membrane proteins. These regions may be associated with serovar differences, considering that Nascimento et al. (2004) already described genome variations due to transposition of mobile genetic elements between *L. interrogans* serovars.

**Fig. 1:**
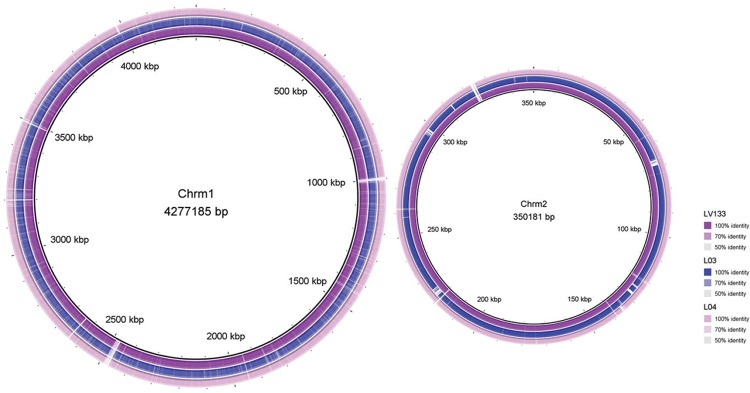
BLAST ring image generator (BRIG) plot displaying whole genome comparison of *Leptospira interrogans* serovars Copenhageni (internal reference line) and Canicola.

Both human and porcine serovar Canicola isolates present the major *Leptospira* virulence factors: lipoproteins LipL32 and LipL41; immunoglobulin-like factors LigA, LigB and LigC; flagelin; OmpA-like protein Loa22. These presented 100% identity with pathogenic *L. interrogans* serovar Copenhageni strain Fiocruz L1-130 genes. Therefore, the porcine serovar Canicola strains present the genetic machinery to cause human infection and represent a higher risk to public health.

The whole genome single nucleotide polymorphism (SNP) analysis was performed with CSI Phylogeny (Kaas et al. 2014) using *L. interrogans* serovar Copenhageni strain Fiocruz L1-130 as reference. For the phylogeny, the Maximum Likelihood method was used based on the general time reversible model. The SNP analysis, considering a pool of 24093 SNPs, enabled further differentiation between human and porcine serovar Canicola isolates despite their great similarity (Fig. 2). The porcine serovar Canicola isolates presented 96% of SNP identity distancing from the human serovar Canicola LV133 strain (91.5 to 92.6% identity).

**Fig. 2:**
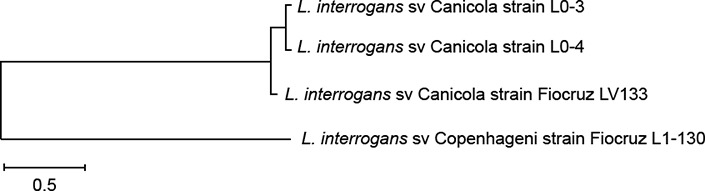
single nucleotide polymorphism (SNP) analysis of *Leptospira interrogans* serovar Canicola human (LV133) and porcine (L0-3 and L0-4) isolates, using serovar Copenhageni strain Fiocruz L1-130 as reference.

Regarding plasmidial content, all sequences similar to the pGui plasmids present GC content < 34% differing from chromosomal content, as previously described (Zhu et al. 2014). Despite the high similarity, the Brazilian pGui1 homologous sequences present size variations (Fig. 3A). However, the main difference between serovar Canicola isolates is observed in the sequence similar to pGui2. Porcine isolates present plasmid sequences ~11.2 kb longer than the original pGui2 and LV133 plasmid (Fig. 3B), corresponding mostly to transposases and AAA ATPase domain proteins related to replication and recombination process.

**Fig. 3:**
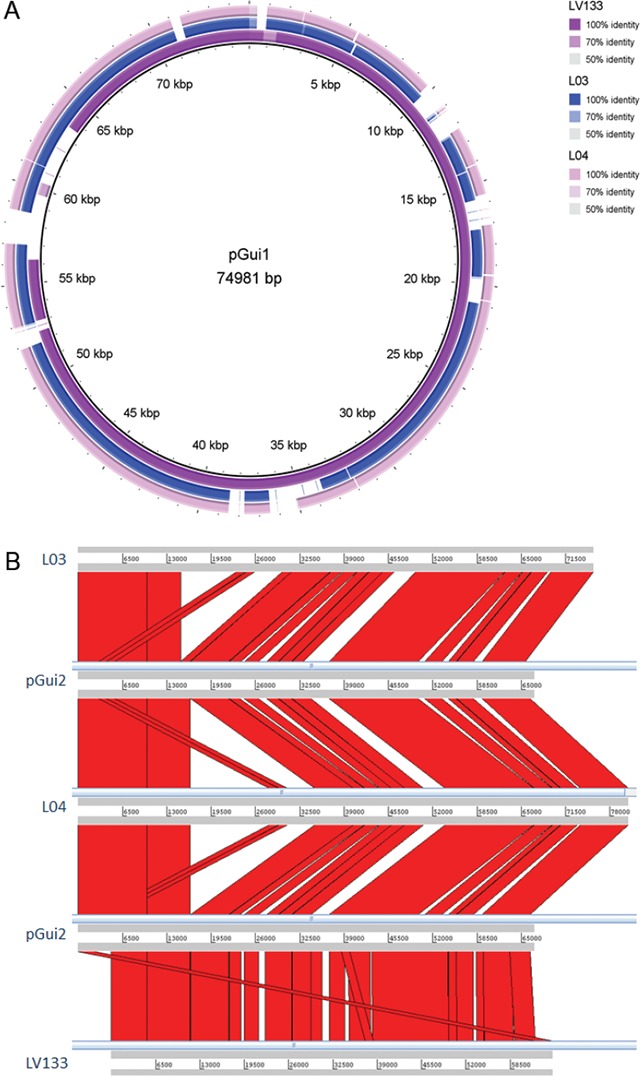
comparative analysis of extrachromosomal sequences homologs to plasmids pGui1 and pGui2. (A) Circular representation of the extrachromosomal element similar to pGui1. (B) Artemis comparison tool (ACT) synteny visualisation of extrachromosomal element similar to pGui2.

The plasmid identification in the Brazilian serovar Canicola strains, as well as the Zhu et al. (2014) original description, suggest that extra-chromosomal elements are one more feature of this serovar that was previously unnoticed. The differences observed between plasmid sequences could be related to isolates origin (hosts and geographical) or even with the fact that L0-3 and L0-4 strains have been maintained in culture medium through multiple passages for the last ten years.

Considering that Lehmann et al. (2016) described *L*. *interrogans* serovar Lai *in vitro* culture-passage attenuation due to cumulative genome changes, mainly virulence genes mutations, the continued maintenance of the studied strains in EMJH medium could have significantly altered not only their chromosomal content, but also the plasmid sequences. Thus, *L. interrogans* serovar Canicola plasmids should be further studied to confirm if their differences are related to host specificities or environmental adaptions.

Nonetheless, the isolation of the serovar Canicola in pigs stands out in Brazil. Although serovars are not entirely unique to the host species, these usually present a certain preference (Schuller et al. 2015). Thus, the epidemiological implications of isolating serovar Canicola from pigs demands attention, with the possibility of *Leptospira* dissemination and maintenance on swine farms by dogs. The high similarity of *L. interrogans* serovar Canicola animal strains with the genome of the human strain also indicates that the porcine strains may pose a greater risk to public health than expected.
